# The Diseases and Deformities of the Fœtus

**Published:** 1893-09

**Authors:** 


### Reviews of Books.

*The Diseases and Deformities of the Fœtus.* By J. W. Ballantyne, M.D. Vol. I. Pp. xiii., 252. Edinburgh: Oliver & Boyd. [1892.]

When so great a demand is made upon our time for acquiring information in the varied departments of medical knowledge, the study of fœtal diseases does not seem an urgent necessity, and yet in it we may find the explanation of post-natal morbid conditions. It is a very difficult and complicated study, for we have to deal not only with fœtal abnormalities, but also with possible morbid states of the mother in relation to them.

Dr. Ballantyne's book will be, we have little doubt, a standard work of reference on the subject. Only the first volume is now published. It contains a chapter on the interest and importance of the subject, another on the history of the study of fœtal diseases, and then we come to the "first great division of the work, the diseases properly so called of the fœtus." We are told that in the second volume the remaining idiopathic, and some of the transmitted, morbid states will be dealt with, and in the third volume the consideration of the diseases proper of the fœtus will be brought to a conclusion. Beyond this point Dr. Ballantyne says "he dares scarcely look, but the remainder of the work will be occupied with the deformities of the fœtus."

Dr. Ballantyne claims that his book is the first which has been written at any length on the diseases and deformities of the fœtus. He however refers to a book by Henry Madge, *The Diseases of the Fœtus in Utero*, published in London in 1854. This work is interesting chiefly from its date, as showing how many years ago some attention was paid to the subject.

We quite agree with Dr. Ballantyne when he says, speaking of the study of fœtal diseases, "it is not just to stamp the whole subject as visionary and impractical." We are sure that the study of fœtal pathology may throw much light on the diseases of infancy and childhood; but we are inclined to think, on the other hand, that perhaps too hopeful a picture is drawn of the advantages to the fœtus itself which may result from this study. We cannot diagnose these diseases and deformities of the fœtus *in utero*, much less treat them, unless, as in the case of syphilis, we suspect them from conditions in the parents. The various forms of clubfoot, perhaps the most interesting of all fœtal abnormalities to the surgeon, cannot be diagnosed *in utero*, and therefore the malposition on which they are now generally supposed to depend cannot be rectified.

Then again the study of fœtal pathology, though important and interesting, must be very difficult and unsatisfactory. As an example let us take Dr. Ballantyne's concluding remarks on fœtal dropsy. He says: "Whilst in some cases of fœtal dropsy there existed an adequate cause in the fœtus itself, in most cases the disease was due to a chain of factors having a cachectic state of the mother at one end, and a blood disease of the fœtus at the other, with a morbid state of the uterine mucosa and placenta intervening." And Dr. Ballantyne himself tells us "that the study of this disease, in common with all fœtal maladies, presents very special difficulties to the investigator; for comparatively little is yet known with regard to the effects of maternal, and to some extent of paternal, morbid states upon the health and development of the fœtus." This is not encouraging to would-be investigators; but perhaps we should not despair of gaining a little exact knowledge after much laborious investigation and a rational scepticism as to deduction on an insufficient basis.

So much for the study of fœtal diseases. With regard to the book, we must say it is a work of great value, or rather will be when it is completed. Its production will be a task of a very laborious kind, if the volumes yet to appear are as thoroughly good as the present one.

